# Rheumatoid Lung Nodules Presenting With Hemoptysis and Cough

**DOI:** 10.7759/cureus.90204

**Published:** 2025-08-16

**Authors:** Ana Oliveira, Rita Figueira, Tatiana Rodrigues, Álvaro Ferreira, Fátima Farinha

**Affiliations:** 1 Internal Medicine, Unidade Local de Saúde da Região de Aveiro, Aveiro, PRT; 2 Internal Medicine, Centro Hospitalar Universitário de Santo António, Porto, PRT; 3 Clinical Immunology Unit, Centro Hospitalar Universitário do Porto, Porto, PRT

**Keywords:** cough, hemoptysis, methotrexate, rheumatoid arthritis, rheumatoid lung nodules

## Abstract

A 64-year-old man, smoker, was diagnosed with seropositive rheumatoid arthritis (RA) for 13 years. He was being treated with methotrexate (MTX) and hydroxychloroquine. He presented to the emergency department due to coughing and hemoptysis over the past month. Chest X-ray revealed a nodular lesion in the right lung. Chest CT scan identified several pulmonary nodules in both lungs and a right hydropneumothorax. Cultures were negative for any infectious causes. Histology of pulmonary nodules favored a diagnosis of rheumatoid lung nodules. He stopped MTX and started baricitinib.

## Introduction

Pulmonary rheumatoid nodules are a rare complication of rheumatoid arthritis (RA). Their prevalence ranges from <0.4% in radiological studies to 32% in lung biopsies of patients with RA and nodules [1]. Those who have an increased risk of lung nodulosis include smokers, male patients with positive rheumatoid factor, patients with subcutaneous nodules, and those on long-term methotrexate (MTX) treatment. Lung nodules are usually multiple and rounded, preferentially located in the middle and superior peripheral lobe or pleural based. Up to 50% may present with pleural effusion, pneumothorax or hydropneumothorax [1]. Pulmonary nodulosis has been shown to be accelerated by MTX. On the other hand, nodules may also be induced by leflunomide, azathioprine, and antitumor necrosis factor (anti-TNF) [2].

## Case presentation

A 64-year-old man, smoker, was diagnosed with seropositive RA in 2007. He presented with joint involvement and cutaneous nodulosis. He was under treatment with a combination of MTX and hydroxychloroquine.

**Figure 1:**
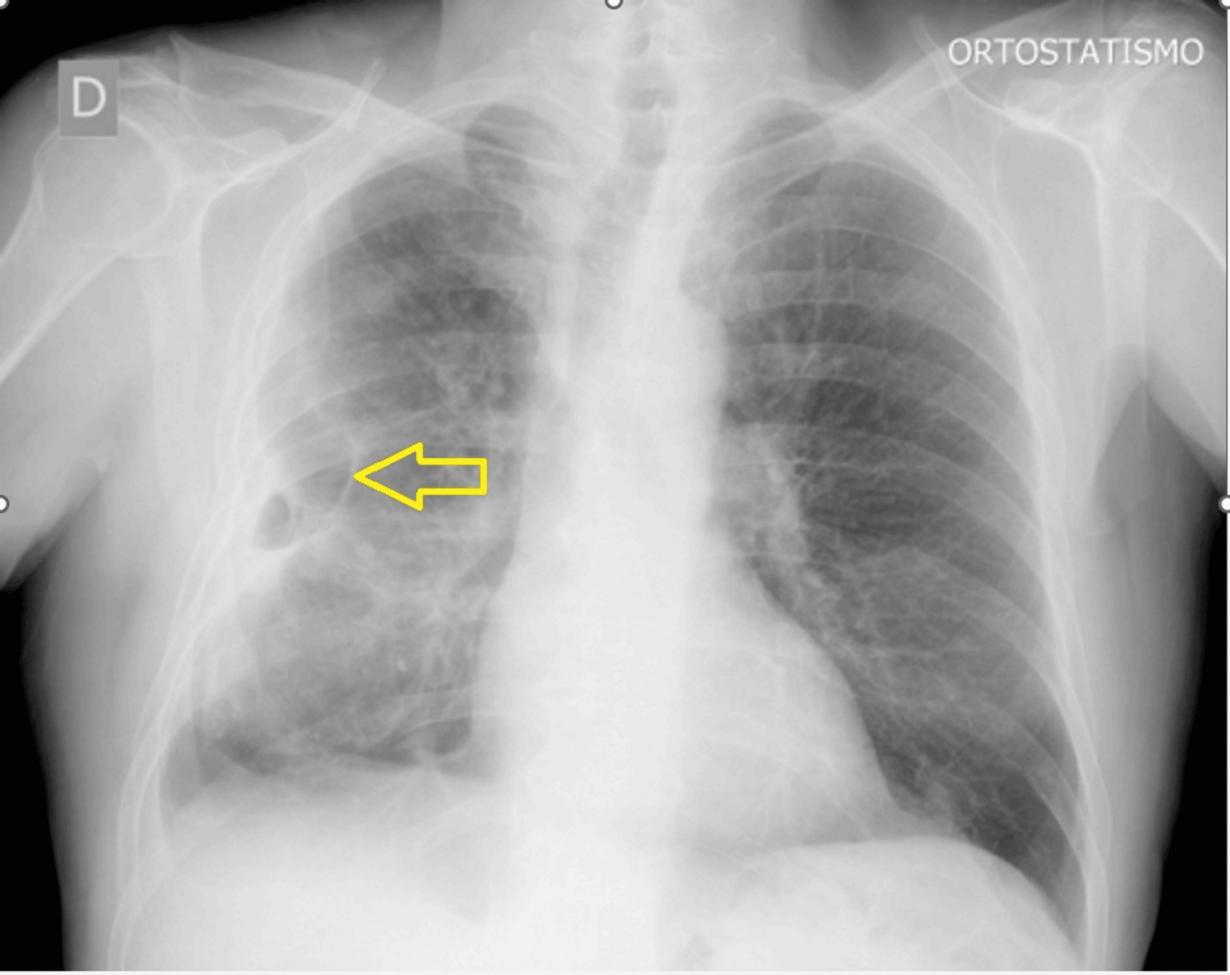
Chest X-ray in posteroanterior incidence The image shows nodular image on the right lung (yellow arrow), which might have been infectious etiology or neoplasm. We also identified a slight effacement of costophrenic sinuses, which was suspected to be a pleural effusion. No others changes were noted.

In August 2020, he presented to the emergency department with a one-month history of cough and hemoptysis. There was no previous history of shortness of breath, fever, malaise, or weight loss. He denied any contact with sick patients. On physical examination, his oxygen saturation was 96% while breathing room air without other alteration. Laboratory reports revealed normal blood count with a C-reactive protein (CRP) of 57.58 mg/L. Chest X-rays revealed a lung nodule on the right side (Figure 1). 

**Figure 2:**
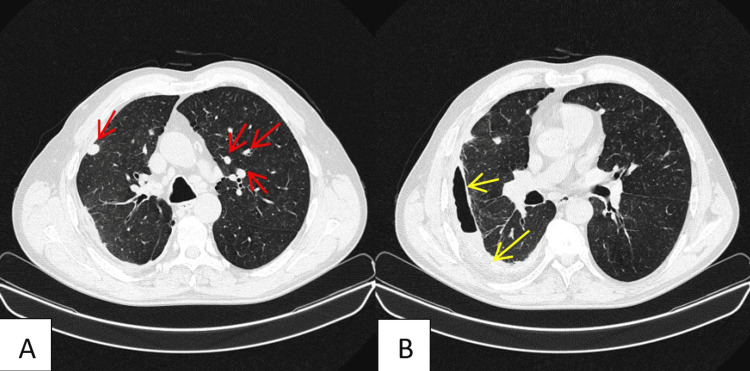
Chest CT scan A: Chest CT showed multiple solid nodules in both lungs (red arrows). These were distributed centrally and peripherally, but most of them were subpleural located. The largest nodules are located in the anterior segment of the left upper lobe, measuring 2.4 cm, and in the right lower lobe, measuring 2.2 cm. B: Right hydropneumothorax (yellow arrows) and mild parenchymal atelectasis are observed in the right lung. They are subpleural, adjacent to the effusion. The parenchyma presents a mild mosaic density pattern, suggesting ventilation-perfusion alterations. No new changes are identified in the lung parenchyma.

Chest CT showed multiple solid nodules in both lungs. These were distributed centrally and peripherally, but most of them were subpleural located (Figure 2A). Right hydropneumothorax and mild atelectasis of the parenchyma in the subpleural right lung, adjacent to the effusion, were also identified (Figure 2B). 

In the mediastinum, multiple lymph nodes were identified to be scattered throughout the different compartments, the largest in the right pulmonary hilum, measuring 11 mm in short axis, and in the subcarinal space, measuring 11 mm in short axis, which were nonspecific.

Lung biopsy showed a granuloma formation with central fibrinoid necrosis surrounded by layers of palisading macrophages and lymphocytes, with no evidence of malignancy. Cultures did not grow fungus or mycobacteria. 

A diagnosis of rheumatoid nodules was made by the clinical setting, typical radiographic, histopathology features, and negative cultures. The patient thus discontinued MTX and started baricitinib to maintain control disease and avoid the progression of pulmonary nodules.

## Discussion

RA is a systemic disease in which nodule formation is well known, though these are predominantly cutaneous. Pulmonary nodulosis is rare and is usually asymptomatic. Symptoms associated with pulmonary nodulosis are cough, dyspnea, and hemoptysis, which are symptoms that can be found in other etiologies. That said, when we have a patient with RA and the respiratory symptoms described above, it is mandatory to exclude other entities, such as infection and neoplasia [2,3].

This case illustrates pulmonary involvement of rheumatoid nodules in a patient with RA, who presented with cough and hemoptysis. A microbiological study of sputum culture was performed, in order to exclude infection. Due to the history of smoking, imaging examination (X-ray and CT) was also performed to observe the lung parenchyma to exclude nodules and parenchymal disease. A biopsy was performed to identify the etiology of the nodule, which was suggestive of a rheumatoid pulmonary nodule.

The appearance and progression of rheumatoid nodules is a consequence of the disease, but can also be triggered by some medications, such as immunosuppressor agents used in the treatment of RA [2-4].

In 1986, Kremer and Lee observed an acceleration in the progression of rheumatoid nodules during a study of prolonged therapy with MTX [4]. This phenomenon was observed by other authors in approximately 10% of RA patients treated with MTX [2,5]. Other drugs, such as leflunoamide, azathioprine, and antitumor necrosis factors (anti-TNFs), have been shown to induce nodulosis [2-5].

Since our patient was on MTX and presented with symptomatic pulmonary rheumatoid nodules, it was necessary to suspend MTX therapy and start a drug for RA that is not associated with nodulosis progression.

In 2018, Venerito et al. reported a regression of a pulmonary nodule after four months of baricitinb treatment. The study reported that Janus kinase inhibition could be an effective treatment for patients with pulmonar rheumatoid nodulosis [6]. More recent study confirm the results obtained by Venerito et al. [7]. Its results highlights the role of baricitinib in the treatment of patients with RA and pulmonary nodulosis.

Our patient started baricitinib and discontinued MTX; he continued taking hydroxychloroquine. A few weeks after starting baricitib, the hemoptysis resolved and the frequency of coughing improved. After six months of treatment, the patient remained asymptomatic. At this time, he underwent a new chest CT scan, which showed a decrease in the size of the nodules in the right lower lobe, and the remaining nodules were stable.

## Conclusions

In conclusion, rheumatoid lung nodules may be related to RA or the drugs used to treat it. Differential diagnosis should be made by excluding infectious and neoplastic causes, since their symptoms are similar, and so are their appearances on imaging. A biopsy is mandatory for diagnosis, as this is the only way to identify nodulosis etiology. Treatment of pulmonary nodules will change the basal treatment of RA to another one that has a lower risk of pulmonary nodulosis.
